# Characterization and Mathematical Modeling of Alginate/Chitosan-Based Nanoparticles Releasing the Chemokine CXCL12 to Attract Glioblastoma Cells

**DOI:** 10.3390/pharmaceutics12040356

**Published:** 2020-04-14

**Authors:** Suzanne Gascon, Angéla Giraldo Solano, Wiam El Kheir, Hélène Therriault, Pierre Berthelin, Bettina Cattier, Bernard Marcos, Nick Virgilio, Benoit Paquette, Nathalie Faucheux, Marc-Antoine Lauzon

**Affiliations:** 1 Laboratory of Cell-Biomaterial Biohybrid Systems, Department of Chemical and Biotechnological Engineering, Faculty of Engineering, Université de Sherbrooke, 2500 boul universite, Sherbrooke, QC J1K 2R1, Canada; Suzanne.Gascon@USherbrooke.ca (S.G.); Pierre.Berthelin@USherbrooke.ca (P.B.); Nathalie.Faucheux@USherbrooke.ca (N.F.); 2 Department of nuclear medicine and radiobiology, Faculty of Medicine and Health Sciences, Université de Sherbrooke, 12e avenue Nord, Sherbrooke, QC J1H 5N4, Canada; Angela.Giraldo.Solano@USherbrooke.ca (A.G.S.); Helene.Therriault@USherbrooke.ca (H.T.); 3 Advanced dynamic cell culture systems laboratory, Department of Chemical and Biotechnology Engineering, Faculty of Engineering, Université de Sherbrooke, 2500 boul universite, Sherbrooke, QC J1K 2R1, Canada; Wiam.El.Kheir@USherbrooke.ca (W.E.K.); b.cattier@hubebi.com (B.C.); 4 Department of Chemical and Biotechnology Engineering, Faculty of Engineering, Université de Sherbrooke, 2500 boul universite, Sherbrooke, QC J1K 2R1, Canada; Bernard.Marcos@USherbrooke.ca; 5 Department of chemical engineering, Polytechnique Montréal, Montréal, QC H3C 3A7, Canada; Nick.Virgilio@polymtl.ca; 6 Department of nuclear medicine and radiobiology, Faculty of Medicine and Health Science, Université de Sherbrooke, 12e avenue Nord, Sherbrooke, QC J1H 5N4, Canada; Benoit.Paquette@USherbrooke.ca; 7 Clinical Research Center of the Centre Hospitalier Universitaire de l’Université de Sherbrooke, 12e avenue Nord, Sherbrooke, QC J1H 5N4, Canada; 8 Research Center on Aging, 1036, rue Belvédère Sud, Sherbrooke, QC J1H 4C4, Canada

**Keywords:** nanoparticles, chitosan, alginate, delivery system, mathematical modeling, chemokine, cell migration

## Abstract

Chitosan (Chit) currently used to prepare nanoparticles (NPs) for brain application can be complexed with negatively charged polymers such as alginate (Alg) to better entrap positively charged molecules such as CXCL12. A sustained CXCL12 gradient created by a delivery system can be used, as a therapeutic approach, to control the migration of cancerous cells infiltrated in peri-tumoral tissues similar to those of glioblastoma multiforme (GBM). For this purpose, we prepared Alg/Chit NPs entrapping CXCL12 and characterized them. We demonstrated that Alg/Chit NPs, with an average size of ~250 nm, entrapped CXCL12 with ~98% efficiency for initial mass loadings varying from 0.372 to 1.490 µg/mg NPs. The release kinetic profiles of CXCL12 were dependent on the initial mass loading, and the released chemokine from NPs after seven days reached 12.6%, 32.3%, and 59.9% of cumulative release for initial contents of 0.372, 0.744, and 1.490 µg CXCL12/mg NPs, respectively. Mathematical modeling of released kinetics showed a predominant diffusive process with strong interactions between Alg and CXCL12. The CXCL12-NPs were not toxic and did not promote F98 GBM cell proliferation, while the released CXCL12 kept its chemotaxis effect. Thus, we developed an efficient and tunable CXCL12 delivery system as a promising therapeutic strategy that aims to be injected into a hydrogel used to fill the cavity after surgical tumor resection. This system will be used to attract infiltrated GBM cells prior to their elimination by conventional treatment without affecting a large zone of healthy brain tissue.

## 1. Introduction

Polymeric-based nanoparticles (NPs) have generated a lot of interest because of their potential as drug delivery systems that can transport molecules across physiological barriers such as the blood-brain barrier [1]. For example, chitosan-based NPs are employed in various tissues or organs, such as the brain, bone, gastrointestinal tract, and cornea to name a few, in order to favor regeneration or to treat disease and infection [2,3,4]. Indeed, chitosan is a biodegradable polysaccharide considered as biocompatible with low immunogenicity and lack of toxicity, and it possesses antitumor, antifungal, and antibacterial activities [5]. It is obtained after partial deacetylation of the chitin, a poly (β-(1→4)-N-acetyl-D-glucosamine), found in crustacean shells (crabs and prawn), the cocoon of insects, and fungi cell wall [6,7,8]. However, due to its cationic and alkaline nature, chitosan must be modified to bear carboxyl groups or combined with negatively charged polymers such as alginate to form NPs that can better entrap positively charged molecules [9,10,11].

CXCL12 (CXC-motive chemokine ligand 12) also known as stromal-cell derived factor 1 (SDF-1) is a positively charged molecule at physiological pH (isoelectric point about 10) with a molecular weight of about 8 kDa [12]. It acts on cells through binding to its main receptor CXCR4. It is involved in stem or progenitor cell migration during tissue regeneration and repair [13,14,15]. CXCL12 also plays a crucial role in tumor proliferation and invasion as well as in the metastasis of cancerous cells such as those of breast cancer or glioblastoma multiforme (GBM) [16,17,18]. GBM is the most frequent and aggressive primary brain tumor in adults leading to a post-diagnosis survival time around 15 months and a 5-year relative survival rate lower than 5% [19,20]. GBM tumor recurrence is mainly due to the migration of GBM cells into the normal brain parenchyma, which limits conventional cancer therapy efficiency (surgical resection and radiotherapy) [21]. To overcome this challenge, a proposed strategy consists in reversing the migration direction of GBM cells using a chemoattractant gradient and concentrate them to a location where they can be eliminated by conventional treatment without affecting the healthy brain tissue. Several studies have therefore targeted CXCL12 or its CXCR4 receptor in order to control normal or cancerous cell migration [22,23]. For example, Rubin et al. demonstrated that soluble CXCL12 increases the migration of U87 GBM cells in a dose-dependent manner [23].

Nevertheless, due to its short half-life of about 26 min in rat blood (radiolabeled CXCL12 using ^99m^Tc-S-acetylmercaptoacetyltriserine) and rapid clearance [24], CXCL12 must be protected by entrapment into NPs upon its local sustained release [9,25,26]. However, the kinetics of CXCL12 released from NPs delivery systems are rarely characterized using mathematical modeling. Such an experimental approach is important not only to better understand the phenomena that control the chemokine delivery but also help to optimize and calibrate the delivery to the desired parameters [10,27]. For example, we have previously developed a composite NPs delivery system made of alginate (Alg) and chitosan (Chit) to encapsulate a positively charged peptide derived from bone morphogenetic protein-9 (SpBMP-9, 23 residues) for brain application. We found that the presence of Alg (negatively charged), besides being essential for the synthesis of NPs by ionotropic gelation, could promote high encapsulation efficiency, strongly enhance the interactions with the peptide, and therefore allow its sustained release for up to 7 days [10]. In the same study, we also developed a mechanistic mathematical framework, adapted to NPs delivery system, to study the release kinetics. This model takes into account the electrostatic interactions between the solute and the polymeric matrix composing the delivery system within the boundary conditions as well as the size distribution of the NPs. This mathematical model allowed us to thoroughly study the mechanisms driving the delivery, which in return could provide essential cues to adapt and optimize the system. This kind of experimental approach is therefore particularly suitable for the encapsulation of CXCL12 for applications targeting the brain tissue. As GBM invasiveness can vary one patient to another, such a delivery system where the delivery can be tuned is thus required.

In the present study, we have therefore prepared and characterized composite Alg/Chit NPs to entrap CXCL12, which upon its release can increase the migration of GBM cells. We have determined the percentage of chemokine encapsulation in Alg/Chit NPs at various initial mass loading using CXCL12 labeled with AlexaFluor^®^ 647 and analyzed its release kinetics. Next, the delivery system was mathematically modeled, and the effective diffusion coefficient and the mass transfer coefficient at the surface were determined. Finally, the toxicity of NPs was evaluated and the efficiency of released CXCL12 from NPs to act on GBM cells migration was also assessed. We demonstrated that Alg/Chit NPs have a high encapsulation efficiency range, are not cytotoxic, and promote GBM cell migration. Mathematical modeling also revealed that, depending on the initial mass loading of CXCL12, the release kinetic can be tuned, which confirmed the pertinence of this delivery system for future GBM treatment. The delivery system aims to be injected into a hydrogel placed in the brain cavity, following the tumor resection in order to attract GBM cells that have invaded the surrounding peri-tumoral tissue and favor their elimination by radiotherapy.

## 2. Materials and Methods 

### 2.1. Materials

High molecular weight (HMW) chitosan (>310 kDa) with a deacetylation percentage of 85%, calcium chloride (CaCl_2_) and cell dissociation solution were purchased from Sigma (Sigma-Aldrich, Oakville, ON, Canada). Sodium alginate with a guluronic acid (G) to mannuronic acid (M) ratio of 2:1 (determined by NMR) with a molecular weight of ~110 kDa (determined by GPC) was kindly offered by Kimica inc. (Tokyo, Japan). Human recombinant CXCL12 (SDF-1α) (purity ≥98%, PeproTech USA, Rocky Hill, NJ, USA) was reconstituted in sterile milliQ water. AlexaFluor^®^ 647 conjugated-CXCL12α (CXCL12-AF647) was purchased from Almac Group (Craigavon, UK) and reconstituted following the manufacturer’s instruction. Cell culture medium (DMEM), trypsin-EDTA (0.25%) and penicillin-streptomycin (10,000 U/mL) antibiotics were purchased from Gibco (ThermoFisher, Burlington, ON, Canada), whereas fetal bovine serum (FBS) was purchased from Wisent Bioproduct Inc. (St-Bruno, QC, Canada). Live (Calcium AM)/dead (ethidium homodimer-1) mammalian cell viability/cytotoxicity kit and Hoechst 33342 staining solution were purchased from ThermoFisher Canada (Burlington, ON, Canada), whereas MTS cell viability/cytotoxicity assay kit was purchased from Promega North America (Madison, WI, USA). Finally, invasion chambers were purchased from Fisher Scientific Ltd. (Toronto, ON, Canada).

### 2.2. Nanoparticle Synthesis

Alg/Chit NPs were synthesized using an ionotropic gelation method as previously reported [10]. Briefly, a CaCl_2_ solution (18 mM) with a final ratio Alg: CaCl_2_ of 5:1 (*w/w*) was added dropwise to a solution of Alg (1 mg/mL, pH 5.1) under vigorous stirring. For experiments involving CXCL12 or CXCL12-AF647, various concentrations of chemokine were added to the Alg solution prior to the addition of CaCl_2_ solution under constant stirring for 5 min in order to favor electrostatic interactions. After 30 min of agitation, an HMW Chit solution (1 mg/mL in 1% (*v/v*) glacial acetic acid) was added dropwise with a final Alg: Chit (*w/w*) ratio of 4:1 and resulted in an opalescent solution containing composite Alg/Chit NPs. The ratio of Alg/CaCl_2_/Chit used is based on the work of Haque et al., who showed that this specific composition led to optimal Alg/Chit NPs formulation with a size range of ~150 to 250 nm and maximum encapsulation efficiency for water soluble and positively charged solute [28]. Alg and Chit properties (MW, G:M ratio) were similar to those we used in the present study. The newly formed Alg/ChitNPs were kept under constant stirring overnight at 4 °C to standardize the size distribution. The NPs solution was then centrifuged at 20,000× *g* (10 °C) for 30 min. The supernatant was kept to determine the CXCL12 encapsulation efficiency.

### 2.3. Size Distribution and Morphology Analysis

The size distribution of freshly prepared Alg/Chit NPs resuspended in deionized water at room temperature was evaluated by laser diffraction (laser granulometry) using a Malvern Mastersizer 2000 (Malvern Instruments Canada, Montreal, QC, Canada) having a liquid dispersion unit. This technique employs low-angle laser light scattering combined with backscattering to determine the particle size distribution (0.02 to 2000 µm) based on Fraunhofer and Mie scattering theories. Alg/Chit NPs were dispersed using ultrasound (low energy). The morphology analysis was assessed by scanning electron microscopy (SEM) observation. Briefly, 20 µL of Alg/Chit NPs solution (diluted 1:5 in pure deionized water) with or without CXCL12 was spread on a sample holder and allowed to completely dry under the biological safety cabinet. Subsequently, the samples were metalized with a coating of gold/palladium. High-resolution images were taken at 30 kV using an S4700-Hitachifield emission scanning electron microscope (Hitachi High-Technologies Canada, Toronto, ON, Canada).

### 2.4. Encapsulation Efficiency and Release Kinetics

Encapsulation efficiency and release kinetics were determined by fluorescence quantification using a microplate reader (Safire2, Tecan US Inc., Morrisville, NC, USA) with an excitation wavelength of 650 nm and an emission wavelength of 665 nm. The corresponding concentration was calculated using a standard curve made of soluble CXCL12-AF647 (0 to 2 µg/mL). More precisely, freshly prepared Alg/Chit NPs-CXCL12-AF647 were separated into two samples and centrifuge at 20,000× *g* (10 °C) for 30 min. One pellet was used to determine the encapsulation efficiency, whereas the other one was used to assess the release kinetics.

#### 2.4.1. Encapsulation Efficiency

The encapsulation efficiency was determined by two approaches: i) the non-encapsulated CXCL12-AF647 that remained in the supernatant was quantified indirectly, ii) the pellet was dissolved into Tris (10 mM)-EDTA (1 mM) buffer for 20 min at room temperature to directly quantify the encapsulated CXCL12-AF647. The encapsulation percentage was determined as follow:

(1) $$Encapsulation\;\left(\%\right)=100\left(\frac{encapsulated\;mass}{initial\;mass\;loading}\right).$$

#### 2.4.2. Release Kinetics

The remaining Alg/Chit NPs pellet was resuspended into sterile phosphate buffer saline (PBS) (350 µL) in order to mimic physiological conditions and transferred to low binding microcentrifuge tubes to avoid non-specific adsorption. These tubes were then incubated in a humidified cell-culture incubator (Forma Series II, water jacket CO_2_ incubator, Thermo Electron Corporation, Gormley, ON, USA) at 37 °C for different time points between 0 and 168 h under static conditions. After each time point (0; 0.5; 1; 2; 4; 6; 8; 24; 48; 72; 168 h), tubes were centrifuged at 20,000× *g* (10 °C) for 30 min. Supernatants were collected and the fluorescence was quantified. The amount of CXCl12-AF647 released was determined as a percentage of cumulative mass release, as described by the following equation:

(2) $$\frac{M_{t}}{M_{\infty }}=100\left(\frac{\sum_{i=0}^{t}M_{i}}{M_{initial}}\right),$$

where *M_t_* represents the total mass of released CXCL12-AF647 at time “*t*”, *M_i_* the cumulative mass of solute released at time *t* and *M_initial_* the initial loaded mass determined experimentally.

The pellet was then resuspended again with fresh PBS and put back to the incubator for the next sampling. After 168 h of incubation, Alg/Chit NPs were dissolved into Tris (10 mM)-EDTA (1 mM) buffer for 20 min at room temperature and the remaining concentration of CXCL12-AF647 was determined by fluorescence quantification.

### 2.5. Mathematical Modeling and Parameter Estimation

#### 2.5.1. Hypotheses and Constraints

Mathematical modeling was performed, as previously reported [10]. Briefly, the mathematical framework considers the following assumptions:

- The NPs are spherical.
- The CXCL12 is uniformly distributed amongst the NPs volume with a concentration below the saturation (monolithic dispersion).
- NPs are already swollen and do not undergo any erosion within the period of investigation.
- Diffusion is the major mass transport phenomena. Diffusion is considered isotropic in the radial dimension of the NPs. In addition, diffusion is assumed to remain constant through space and time.
- The positively charged CXCL12 (pI of ~10) and the negatively charged Alg chains can undergo electrostatic interactions, which drive the release of the chemokine at the surface of the NPs.

#### 2.5.2. Mathematical Model Formulation

From those assumptions, the main mathematical framework is based on Fick’s second law of diffusion for spherical coordinates with *r* denoting the radius coordinates:

(3) $$\frac{\partial C_{CXCL12}}{\partial t}=\frac{D_{eff}}{r^{2}}\left[\frac{\partial }{\partial r}\left(\frac{\partial C_{CXCL12}}{\partial r}\right)\right].$$

With the following initial conditions:

(4) $$\mathrm{At}\;t=0,\;\forall \;r\;\in \left[0,\;R\right]\to C_{CXCL12}\left(0,r\right)=C_{initial},$$

where *R* represents the total radius of a given NPs.

As we have previously reported [10], we used Newmann boundary conditions, as the mass flux is null at the center of the nanoparticle.

(5) $${\left(\frac{\partial C_{CXCL12}}{\partial r}\right)}_{r=0}=0.$$

We also assumed that the release at the surface of the NPs is driven by the concentration gradient between the chemokine concentration at a given time and the pseudo-equilibrium concentration influenced by the electrostatic interactions between the solute and the NPs. Hence, Robin boundaries conditions were used:

(6) $${\left(\frac{\partial C_{CXCL12}}{\partial r}\right)}_{r=R}=k\left(C_{CXCL12}{}_{R}-C_{eq}\right),$$

where

(7) $$C_{eq}=C_{initial}\left[1-{\left(\frac{M_{t}}{M_{\infty }}\right)}_{eq}\right],$$

where *M_t_* is the cumulative mass release at time *t* and *M*_∞_ is the released mass when time tends to infinity. Here, *M_∞_* is estimated experimentally.

#### 2.5.3. Model Resolution

To solve the mathematical framework, due to the simple morphology of the NPs and the isotropic nature of the release, a finite difference approach was used. More precisely, an implicit Crank-Nicolson scheme was used as it is unconditionally numerically stable. The model resolution also takes into account the size distribution of the NPs. The distribution is partitioned into size classes based on laser diffraction results. The model is then solved for each size class considering a mesh size proportional to the NP diameter as well as the number of NPs belonging to each size class. The time-space solving process can be described by the following matrix system:

(8) $$\forall \;i\in \left[1,\;n_{classes}\right]\;\mathrm{solve}\;C_{i}^{t+1}=A^{-1}\left[BC_{i}^{t}+E\right],$$

where *A* et *B* are tridiagonal matrices corresponding to time step “*t*+1” and time step “*t*”, respectively, and where *E* is a vector carrying information about the boundary conditions.

The total amount of CXCL12 released from all the NPs at each time step (*M_t_*) can be estimated using a trapezoidal method:

(9) $$M_{t}=M_{\infty }-\left[\frac{4\pi }{3}\overset{n_{classes}}{\underset{i=1}{\sum }}N_{particles_{i}}\overset{J_{i}}{\underset{j=0}{\sum }}\left[\left(\frac{C_{j+1,i}^{t}+C_{j,i}^{t}}{2}\right)\left(r_{j+1,\;i}^{3}-r_{j,\;i}^{3}\right)\right]\right],$$

where

(10) $$N_{particles_{i}}=\sum_{j=1}^{n_{classes_{i}}}N_{subclasses\;i,\;j}.$$

#### 2.5.4. Estimation of the Mechanistic Parameters

Then, the diffusion coefficient (*D_eff_*) and the overall mass transfer coefficient at the surface (*k*) were estimated from the experimental data using a genetic algorithm, as previously described [10,29]. The genetic algorithm belongs to the global optimization meta-heuristic algorithm family, which allows for the search of the global optimum in multimodal objective functions. Here, we minimized the sum of squares of residuals between the model and the experimental data, which, using evolutionary algorithms, is defined as the fitness.

### 2.6. Cell Culture

Rat F98 cells (ATCC^®^ CRL-2397TM) were cultured in T75 cell culture-treated polystyrene flasks or 48-well plates (Corning, USA) in DMEM medium supplemented with 1% (*v/v*) penicillin-streptomycin (10,000 U/mL) and 10% (*v/v*) FBS. Cells were grown in 5% CO_2_ in air at 37 °C. The F98 cells were passed to a new culture medium every 3 days.

### 2.7. Viability Assays

F98 cells were seeded into 48-well plates at a density of 28,000 cells/cm^2^ and incubated until they reached 80% of confluence. Cells were then stimulated for 24, 48, and 72 h in cultured medium supplemented with 2% (*v/v*) FBS containing either CXCL12 in solution (0.1, 0.4, and 0.8 µg/mL) or Alg/Chit NPs-CXCL12 at 0.744 µg/mg NPs. Empty Alg/Chit NPs were used as a negative control. After each incubation time, cell survival was evaluated by three approaches: i) enzymatic assay (MTS), ii) nucleus staining followed by cell counting, and iii) live/dead cell viability assay. For the enzymatic assay, MTS reagent (CellTiter 96^®^ aqueous Solution; Promega, Madison, WI, USA) was used according to the manufacturer’s instructions. Briefly, following the incubation, the supernatant was collected, and the absorbance was measured with a spectrophotometer at a wavelength of 490 nm (Safire2, Tecan US Inc., Morrisville, NC, USA).

**Figure 1:**
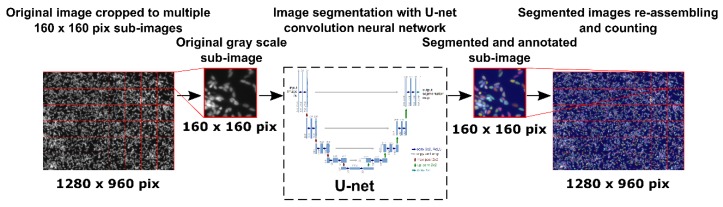
Schematic of the segmentation and counting of cell or nucleus using a U-net convolution neural network.

For cell counting and mortality percentage assessment, cell nuclei were stained with Hoechst 33342 (5 µg/mL) for 30 min or with Live (Calcein AM (2 µM))/Dead (EthD-1 (4 µM)) cell viability kit following the manufacturer’s instruction. Following the staining, 25 stitched-pictures were taken per well by EVOS FL-Auto epifluorescence microscope (Life Technologies, Thermo Fisher, Burlington, ON, Canada) with a magnification of 10×. Stained nuclei and cells stained with live/dead cell viability assay were then counted using a homemade image analysis application. Because F98 cells do not have contact inhibition, they tend to agglomerate and superimpose on top of each other, which makes it difficult to discriminate and count. Prior to count the cells, individual nucleus (Hoechst staining) or cell bodies (Live/Dead staining) were segmented using a U-net convolution artificial neural network using Tensor Flow, which has initially been trained in our laboratory with various cell types and nucleus images (Figure 1). This type of artificial neural network is particularly adapted to cell segmentation [30]. A typical cell counting algorithm was then used (OpenCV library connected component function, with 8-pixel connectivity) to count the number of cells. Cell counting results were then standardized with respect to the image surface using the information (pixels/µm) provided by the microscope software.

For cell mortality assessment, the number of dead cells (cells stained with ethidium homodimer-1 staining) were counted from the live/dead cell viability assay. Cell mortality percentage was determined using the following equation:

(11) $$Cell\;mortality\;\left(\%\right)=100\left(\frac{dead\;cells}{Total\;cell\;number}\right).$$

### 2.8. Invasion Assay

To eliminate endogenous CXCL12, the F98 cells were deprived of FBS by incubating them in DMEM supplemented with 0.1% (*w/v*) bovine serum albumin (BSA) for 24 h at 37 °C and harvested with cell dissociation solution. The F98 cells (4 × 10^4^) were then deposited in the upper compartment of the Boyden invasion chambers, which contains a thin layer of Matrigel. The NPs-CXCL12 were incubated in DMEM 0.1% (*w/v*) BSA for 24 h to allow the release of CXCL12. After centrifugation, 500 µL containing 100 ng/mL of CXCL12 was added in the lower compartment of the invasion chamber. This step makes it possible to evaluate the rate of F98 cell invasion without being affected by the rate of CXCL12 release from the NPs. The F98 cells having crossed the Matrigel layer and the porous membrane 6 h later were fixed, stained, and counted under the microscope. Five controls were performed: 1) DMEM 0.1% BSA; 2) empty NPs; 3) empty NPs 24 h incubation with the extraction of the supernatant + CXCL12 (100 ng/mL) added in the lower compartment of the chamber just before beginning the invasion assay; 4) CXCL12 24 h incubation (100 ng/mL); and 5) CXCL12 0 h incubation (100 ng/mL) added in the lower compartment of the chamber just before beginning the invasion assay. The number of stained cells counted was then reported with respect to the control (DMEM 0.1% BSA), which is defined as the invasion ratio.

### 2.9. Statistical Analysis

Statistical analyses were assessed on Microsoft Excel (Analysis tool pack) by means of the analysis of variance (one way or two-way ANOVA) or GraphPad followed by Tuckey post-hoc pairwise or False Discovery Rate comparison tests. Only differences with *p* < 0.05 were considered statistically significant.

## 3. Results

### 3.1. Nanoparticle Synthesis and Characterization

#### 3.1.1. Size Distribution and Morphology of Alg/Chit NPs

**Figure 2:**
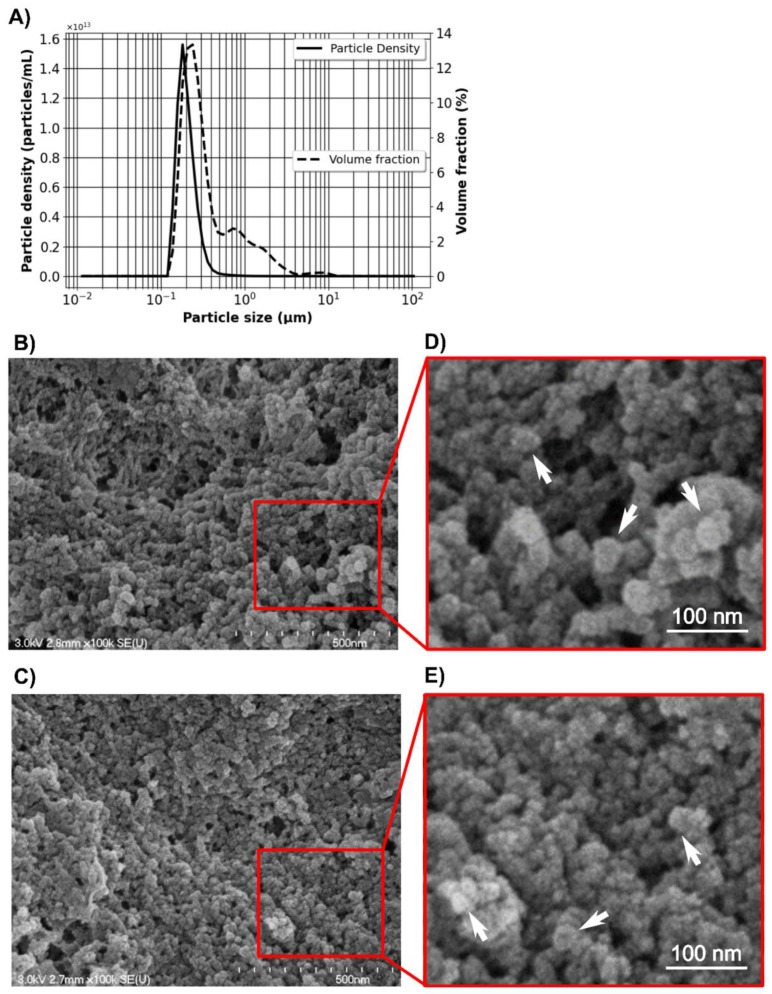
(**A**) Size distribution in volume % and particle density (number of particle/mL) of Alg/Chit NPs obtained from laser granulometry; 5–10 measurements were taken per experiment and the results are representative of three independent experiments. (**B**) Representative SEM images of empty NPs (**C**) and NPs containing CXCL12 (0.744 µg/mg NPs). (**D**) and (**E**) are zoom regions highlighted by red squares for NPs and NPs-CXCL12, respectively. White arrows point to individual NPs.

Alg/Chit NPs underwent a series of characterization steps. NPs were first analyzed for size distribution using laser diffraction (Figure 2A). The results, given as volume fractions, showed a distribution with an average size of ~250 nm in volume (span of 2.89). The distribution was not perfectly bell-shaped since elements with sizes ranging from 500 nm and 5 µm could be observed in the right tail of the distribution. To confirm whether those large particles account for a great number, we then converted the volume fraction to particle density (Figure 2A). The particle density results (number of particle/mL) showed a narrower distribution (span of 0.75) with a diameter range from 100 to 500 nm and an average size of ~207 nm. The differences observed between the volume and density distribution could be explained by the presence of larger aggregates, which have a great influence over the total volume. However, their numbers are negligible. SEM (Figure 2B–E) analyses of dehydrated Alg/Chit NPs confirmed those results. We observed individual solid NPs with a size of ~ 50 nm and a spherical shape (Figure 2B,D). Moreover, we also evaluated the effect of the presence of CXCL12 encapsulated within NPs on their apparent size and shape (Figure 2C,E). The presence of CXCL12 did not seem to affect the relative size of NPs as we have previously observed, which was also reported by other groups working with similar Alg/chit based NPs systems [10,31,32]. The difference in NPs average size between those two techniques reside in the fact that laser diffraction measures NPs in an aqueous environment, whereas scanning electron microscopy uses dehydrated samples. Alg/Chit NPs are known to swell with a ratio of ~400% to 600% [28,33,34,35,36]. In the context of the delivery system, particle size diameter in wet conditions is to be considered, whereas SEM imaging provides information on the particle shape and general impact of drug encapsulation.

#### 3.1.2. Encapsulation Efficiency and Release Kinetics

The capacity of Alg/Chit NPs to encapsulate CXCL12 was evaluated by quantification of CXCL12 remaining in the supernatant following Alg/Chit NPs synthesis. Several initial mass loadings of CXCL12 tagged with a fluorescent dye (AlexaFluor^®^ 647) were assessed, from 0.4 to 1.6 µg/mL (corresponding to 0.372 to 1.490 µg/mg NPs), which are representative of the concentration range required for therapeutic application targeting glioblastoma cells (Table 1). Interestingly, the loading efficiency is quite high (~96.7% to 98.5%) and nearly independent of the CXCL12 initial mass loading. Therefore, increasing the initial mass loading of CXCL12 significantly increased the total encapsulated mass. Those results indicated that the Alg/Chit NPs are an efficient vehicle for CXCL12 encapsulation. CXCL12 encapsulation was also determined by the quantification of the CXCL12 from disrupted NPs (data not shown). The encapsulation efficiency determined by quantifying CXCL12 that remained in the supernatant after the NPs preparation or obtained from the disruption of Alg/Chit NPs was similar.

**Figure 3:**
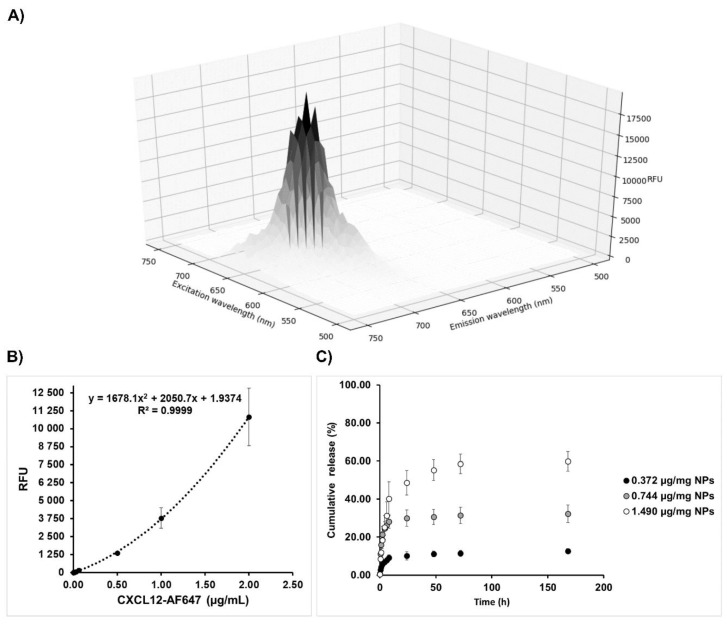
(**A**) 3D fluorescence scan of CXCL12-AF647 (2 µg/mL). (**B**) Standard CXCL12-AF647 curve. **C**) Release kinetics ± SD of CXCL12-AF647 from Alg/Chit NPs for different mass loadings (0.372 µg/mg NPs, 0.744 µg/mg NPs, and 1.490 µg/mg NPs). Results are representative of at least three independent experiments performed in duplicate.

For the CXCL12 release kinetic experiments, several initial CXCL12 mass loadings were used to investigate the effect on the cumulative mass released (Figure 3). The release of CXCL12 from Alg/Chit NPs was quantified over a period of 168 h (7 days), using a CXCL12-AF647 standard curve obtained by fluorescence measurement (Figure 3A,B). For all experimental conditions, an initial burst release was observed during the first 2 h, generally caused by the fast diffusion of the solute located near or at the surface of NPs, followed by a sustained release, which reached a pseudo-plateau (Figure 3C). The sustained release for the 0.372 and 0.744 µg/mg NPs formulation lasts up to 24 h, whereas for the highest initial mass loading (1.490 µg/mg NPs), the pseudo-plateau was reached after 72 h. We also observed that as the initial mass loading increased, the total percentage of cumulative mass release also increased from ~12.8% for 0.372 µg/mg NPs formulation up to ~60% for the 1.490 µg/mg NPs formulation. An increase in the release percentage is coherent with a diffusion-based system, as increasing the initial loading mass generated a higher concentration gradient driving CXCL12 release. However, for all experimental conditions, a pseudo-plateau was reached without a complete release of the CXCL12, which remain stable for up to 7 days (168 h). Those results suggest strong electrostatic interactions, as already observed in previous studies [10,37,38]. In order to validate whether the remaining CXCL12 was still entrapped within the Alg/Chit NPs, NPs were dissociated using a Tris-EDTA buffer after 168 h. We found that the cumulative mass release plus the remaining CXCL12 accounted for ~100% of the total initial mass encapsulated (data not shown).

#### 3.1.3. Mathematical Modeling and Parameter Estimation

To evaluate the driving mass transport phenomenon, we first modeled the release kinetics data (% cumulative mass release) corresponding to the burst and the sustained released (first 8 h of release) using the Korsmeyer-Peppas representation, Equation (12) [39]:

(12) $$\frac{M_{t}}{M_{\infty }}=kt^{n}.$$

To do so, we performed a standard linear regression over the results plot on a logarithmic scale, from which the value of the exponential term “*n*” corresponding to the slope was estimated. The value of the exponential term of the model can provide valuable insights about the governing mass transport phenomena with respect to the geometry of the delivery system [39,40]. The results showed that the model could fit well the experimental data with a coefficient of determination of ~0.98% (Table 2). The value of the exponential term of the model gave values of ~0.4 for all experimental conditions. For spherical geometry, values of exponential term ≤0.43 is indicative of Fickian diffusion as the predominant releasing mechanism [39].

**Figure 4:**
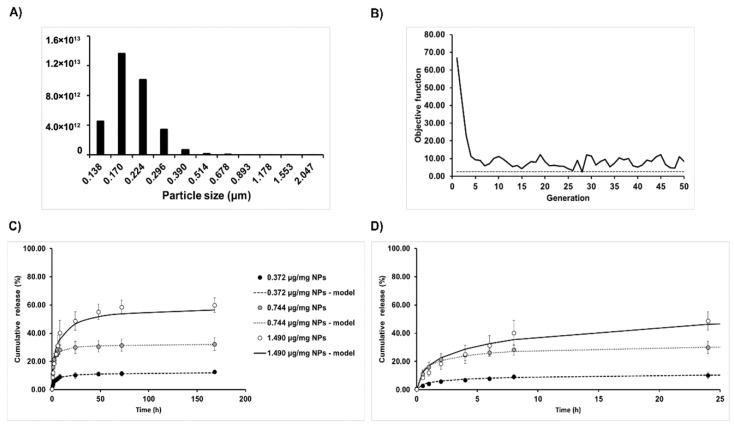
Mathematical modeling of CXCL12 release. (**A**) Particle size classes used in the modeling process adapted from laser diffraction results. (**B**) Representative graphic showing the objective function to minimize with respect to the number of generation performed with the evolutionary optimization algorithm. (**C**) Mathematical modeling of CXCL12 for different initial loading mass. Dashed lines show modeling results. (**D**) Mathematical modeling results for the first 24 h (zoom from **C**).

Since the principal driving force of the release of CXCL12 was diffusion, we used Fick’s second law of diffusion to model the release kinetics with boundary conditions that considered the interactions between the solute and the delivery system. To perform the mathematical modeling, the size distribution of the NPs was also considered (Figure 4A) in order to model the release more faithfully, as we have previously reported [10]. Then, an evolutionary optimization algorithm was used to estimate the values of the effective diffusion (*D_eff_*) and the overall mass transfer coefficient (*k*) with respect to the initial CXCL12 mass loading (Table 3). We have previously shown that this specific model has many local minima and one global minima, which justifies the use of a meta-heuristic algorithm [10].

As shown in Table 3 and Figure 4, respectively, the model parameters, as well as the mathematical framework, was a good fit for all the experimental conditions as shown by a high coefficient of determination (*R*^2^ > ~ 0.97), statistical significance (*p* < 0.001) and non-significant lack of fit. In addition, the optimization algorithm reached its optimal fitness value (minimization of the sum of the square) after 25 to 30 iterations (Figure 4B). Values of the diffusion coefficient (*D_eff_*) were in the same order of magnitude (~1 × 10^−19^) between the experimental conditions, meaning that the diffusion of the molecules appears to be independent of the initial mass loading. As for the overall mass transfer coefficient at the surface (*k*), a decrease in the values of several orders of magnitude was observed as the initial mass loading increased, which indicates that the resistance to the mass transfer at the surface of NPs increases as did the initial mass loading. This means that, at low initial mass loading, the limiting step governing the CXCL12 release rate, which is the diffusion step of the chemokine within the NPs toward their surface, is gradually being replaced by the transfer of CXCL12 between the surface and the surrounding medium (PBS). This was in accordance with the experimental observations where an increase in the initial mass loading resulted in a time shift reaching the pseudo-plateau associated with a slower delivery rate (Figure 4C,D).

### 3.2. Effect of Nanoparticles Releasing the Chemokine CXCL12 on Glioblastoma Cells

#### 3.2.1. Cytotoxicity of Nanoparticles

**Figure 5:**
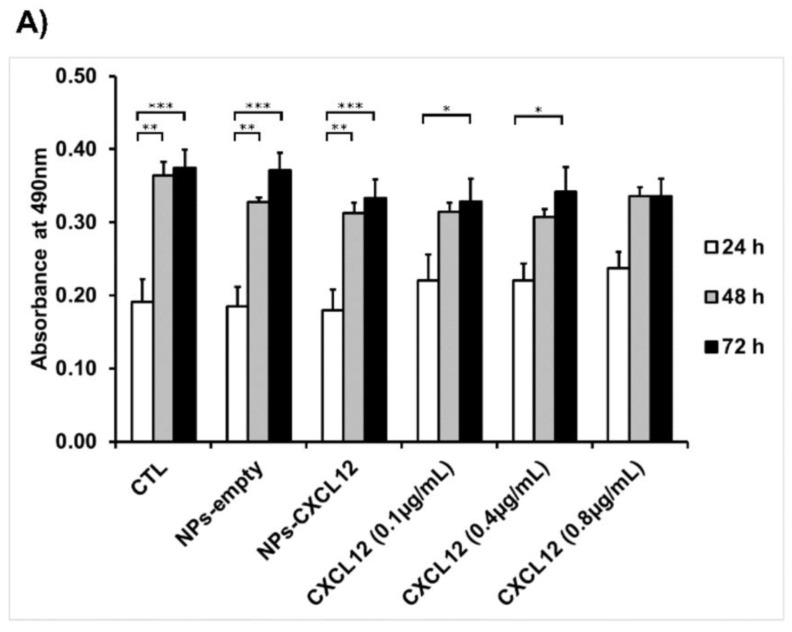

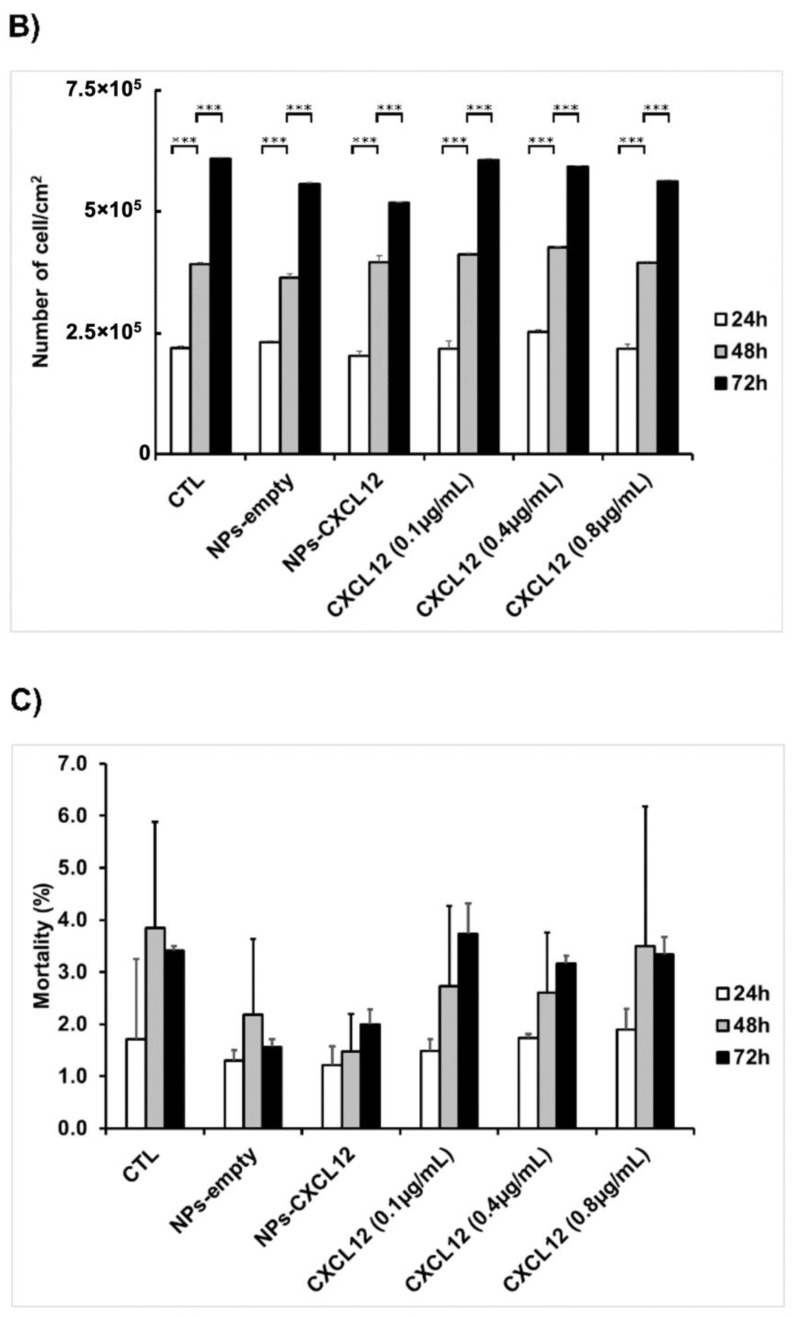
Cytotoxicity of NPs. (**A**) MTS assay results showing the absorbance measured at 490 nm (±SD), (**B**) Kinetic of F98 cell proliferation (±SD). (**C**) Percentage of cell mortality determined from live/dead cell viability assay. Results are representative of three independent experiments performed in duplicate. * *p* < 0.05, ** *p* < 0.01, *** *p* < 0.001.

**Figure 6:**
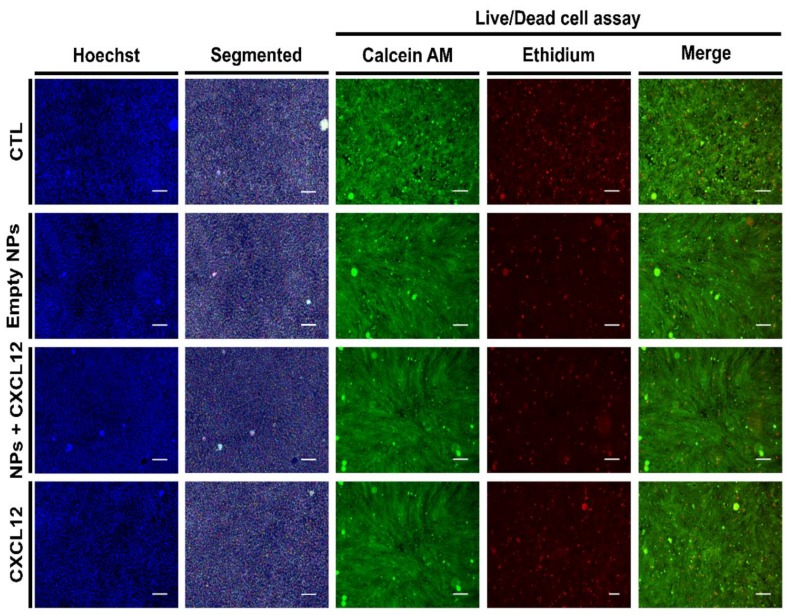
Representative Hoechst, segmented Hoechst (Segmented) and live/dead images for F98 cells incubated for 72 h with empty NPs, NPs-CXCL12 (0.744 µg/mg NPs) and CXCL12 (0.8 µg/mL). Bar = 100 µm. Results are representative of three independent experiments performed in duplicate.

Once the Alg/Chit NPs were characterized, the next step was to assess their effect on GBM cells as they are developed to be used as a delivery system to attract GBM cells following tumor resection. The survival of F98 cells was assessed at the contact of both empty NPs and NPs-CXCL12 (0.744 µg/mg NPs) (Figure 5 and Figure 6). MTS cytotoxicity assay (Figure 5A), which measures the metabolic activity of the mitochondrial enzyme succinate dehydrogenases, revealed that between 24, 48, and 72 h, both empty NPs and NPs-CXCL12 did not have any impact on cell viability as compared to the control (CTL) or various solutions of soluble CXCL12. In addition, direct cell counting (Figure 5B) revealed that no significant difference in F98 cell proliferation was observed for all experimental conditions, as cell density increased similarly from 24 to 72 h. Those results were also confirmed by live/dead cell viability assay (Figure 5C and Figure 6), from which a low percentage of mortality similar between all experimental conditions (~2–5 %) was measured (Figure 5C). Those results indicate that empty Alg/Chit NPs and NPs-CXCL12 do not affect significantly F98 cell viability and proliferation.

#### 3.2.2. Invasion Assay

**Figure 7:**
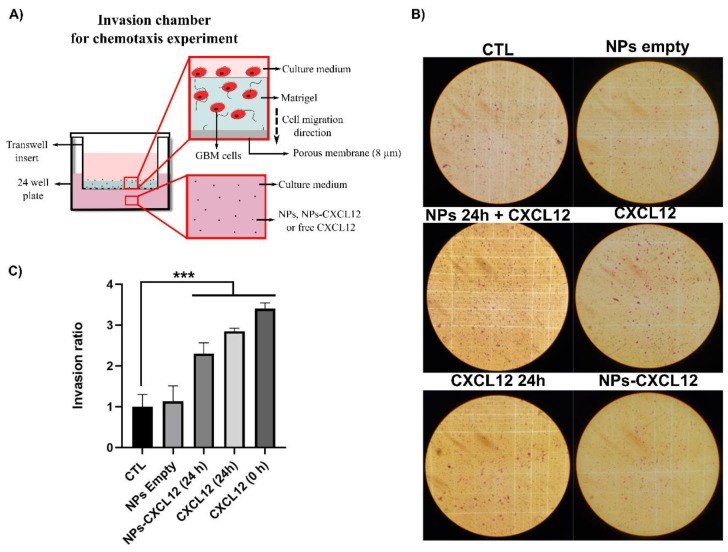
(**A**) Schematic of the invasion chambers used to perform chemotaxis assays. (**B**) Representative images of crystal violet stained F98 cells located at the porous membrane surface following 6 h of incubation for the following conditions: (1) DMEM 0.1% BSA (CTL); (2) empty NPs; (3) empty NPs 24 h incubation with the extraction of the supernatant + CXCL12 added before seeding the F98 cells; (4) CXCL12 24 h incubation; (5) NPs-CXCL12 24 h incubation and (6) CXCL12 0 h incubation. Each condition always had a theoretical final concentration of 100 ng/mL. (**C**) Invasion ratio of F98 cells added on the top of a Matrigel layer and allowed to migrate for 6 h for the following conditions: (1) DMEM 0.1% BSA (CTL); (2) empty NPs; (3) NPs-CXCL12 24 h incubation; (4) CXCL12 24 h incubation, and (5) CXCL12 0 h incubation. Results are representative of three to five independent experiments performed in triplicate. *** *p* < 0.001.

The bioactivity of CXCL12 released from the NPs was then determined by measuring its ability to promote the invasion of F98 cells through a layer of Matrigel (Figure 7A). NPs-CXCL12 corresponding to a final total encapsulation concentration of ~0.1 µg/mL was incubated for 24 h in culture media at 37 °C to allow the release of CXCL12 from the NPs. The supernatant was then collected and added to the lower compartment of the invasion chamber. Soluble CXCL12 (0.1 µg/mL) aged for 24 h, as well as fresh soluble CXCL12 (0.1 µg/mL), were used as positive controls, whereas empty NPs were used as a negative control. Empty NPs were also mixed with fresh soluble CXCL12 (0.1 µg/mL) in order to evaluate their effect on the chemokine in solution. After 6 h, cells that have crossed the layer of Matrigel and the porous membrane were fixed, stained with crystal violet dye and manually counted with a microscope (Figure 7B). Invasion ratio results showed that all experimental conditions involving CXCL12 increased significantly (*p* < 0.001) the F98 cell invasion by ~2.5 for NPs-CXCL12 to ~3.5 times for fresh CXCl12 (0.1 µg/mL) as compared to both empty NPs and control (Figure 7C). Empty NPs incubated with fresh soluble CXCL12 did not significantly modify its ability to attract the F98 cells (results not shown). Those results indicate that CXCL12 released from Alg/Chit NPs was still bioactive and can attract F98 cells. Results also suggest that NPs can prevent CXCL12 from denaturation to a certain extent and do not affect the bioactivity once released.

## 4. Discussion

CXCL12 has emerged as an important factor able to regulate the migration of cancerous cells such as GBM cells [23,41]. GBM cells can infiltrate the normal brain parenchyma, leading to non-curative surgical resection and limiting the impact of therapeutic intervention such as chemotherapy and/or radiotherapy [19]. To overcome this challenge, one possible strategy involves reversing the migration direction of GBM cells by means of a chemokine gradient, such as CXCL12, following tumor resection. Once brought back to the tumor resection site, conventional treatment can be applied to eliminate them without affecting a large zone of healthy brain tissue. Such an approach requires a sustained release of CXCL12 within the brain cavity. A delivery system can act as a shelter for a chemoattractant while controlling its release over space and time. With this strategy in mind, we have developed and characterized a CXCL12 delivery system based on Alg/Chit NPs. NPs made of polyelectrolyte complexes such as Alg/Chit are particularly adapted for addressing molecules to the brain tissue and have potential to treat gliomas [42,43]. They can easily be freeze-dried and reconstituted without affecting the size, release capacity, and bioactivity of the solute contained within [31]. Additionally, NPs remain stable for several hours, even days [32,35]. Those NPs can be further implanted into a hydrogel or a wafer (e.g., Gliadel Wafer^®^) placed inside the brain cavity following tumor resection in order to attract GBM cells that have invaded the surrounding peri-tumoral tissue. The average size of our NPs in aqueous solution was approximately 250 nm. These results were in accordance with our previous study [10]. The CXCL12 did not appear to enhance the size of the NPs. These NPs were in the same size range as those in other studies using Chit-based NPs [25,32]. For example, Wang et al. prepared NPs of various sizes depending on the Chit/heparin volume ratio to encapsulate CXCL12 [25]. While a Chit/heparin volume ratio of 1:5 resulted in an average NPs size of 96 nm, it was increased to 210 nm at a volume ratio of 5:4. Mi et al. also developed Chit-based NPs to deliver CXCL12 with sizes varying from 133 to 297 nm depending on Chit/carboxymethylChit/triphosphate volume ratio [9]. The NPs size is an important parameter since it controls surface area to volume ratio which is involved in several phenomena such as drug delivery or cytotoxicity effect [44]. An increase in the surface area leads to an increase in the release of the solute. Therefore, small NPs will deliver rapidly their content, whereas bigger NPs will deliver it more slowly.

Our results also showed that Alg/Chit NPs were efficient to incorporate a positively charged protein such as CXCL12 since the percentage of CXCL12 encapsulation in the Alg/Chit NPs was higher than 96.7%. Interestingly, the loss of this expensive chemokine during its encapsulation process was very low, despite a four-fold increase in the initial mass loading (from 0.4 to 1.6 µg/mL corresponding to 0.372 and 1.490 µg/mg NPs). Therefore, the CXCL12 can be efficiently entrapped by NPs made of Alg/Chit. Wang et al. have added heparin, a biomacromolecule also rich in carboxylic acid groups that can interact with CXCL12, to prepare Chit-based NPs [25]. Using an initial mass loading of 0.25, 0.5, and 1 µg CXCL12/mg NPs made of Chit/heparin at a volume ratio of 5:4, they obtained encapsulation efficiencies of 93.5%, 94.3%, and 94.8% respectively (94.8% being the maximal value). In the same way, Mi et al. have studied the influence of Chit/carboxymethylChit/triphosphate volume ratio on CXCL12 loading efficacy (LE). Using an initial mass loading of 0.5 µg, they obtained a 65% LE for Chit/carboxymethylChit/triphosphate volume ratio (µL) of 400:100:25, while it reached a maximal value of 85% at a 100:100:25 volume ratio [9]. Mansor et al. have used poly-(lactic-co-glycolic acid) (PLGA)-COOH/polyethylene glycol (PEG)-PLGA NPs with various carboxylic acid groups availability (PLGA-COOH proportion of 0%, 17%, 33%, and 67%), improving the encapsulation efficiency of CXCL12 [26]. Using a precipitate suspension containing 10 µg CXCL12, they obtained 75% of encapsulation in NPs with a PLGA-COOH proportion of 0%. On the other hand, they surprisingly found encapsulation efficiencies above 100% for two PLGA-COOH proportions (17 and 67%) with encapsulations of 104% and 107%, respectively [26]. In the present study, we have been able to prepare, in a reproducible way, Alg/Chit NPs that can entrap CXCL12 at a high encapsulation efficiency without loss of the chemokine despite an increase in initial mass loading up to 1.490 µg/mg of NPs.

Our study also showed that the initial mass loading influenced the release kinetics. After an initial burst effect, a sustained release was observed for all initial mass loadings investigated. However, while for initial CXCL12 mass loadings of 0.372 and 0.744 µg/mg NPs, a pseudo plateau was reached after 24 h, it was observed only after 3 days at a mass loading of 1.490 µg/mg NPs. Furthermore, the percentage of released CXCL12 for initial mass loading of 0.372, 0.744, and 1.490 µg/mg NPs were 12.6%, 32.4%, and 59.9% after seven days, respectively, indicating that the NPs can keep a high dose of this chemokine depending on the initial loaded mass. Since the percentage of encapsulation was similar for all conditions tested, the practical loading of CXCL12 did not reach a plateau within the concentration range used. This explained the release kinetics observed. Mansor et al. have analyzed the release kinetic profile from PLGA-COOH/PEG-PLGA NPs (PLGA-COOH proportion of 0%; NPs prepared using precipitate suspension containing 10 µg CXCL12) and found a burst effect with 50% of CXCL12 released within 12 h. A plateau was reached after 48 h, with more than 75% of encapsulated CXCL12 released [26]. Zamproni et al. also used PLGA NPs containing 106 ng of CXCL12 combined with BSA per mg of NPs for brain application and analyzed the release kinetic profile using ELISA assays. However, they did not observe any initial burst effect since only 5% of CXCL12 was released after 4 h and 30% (cumulative release) was detected after 15 days [45].

The CXCL12 release kinetic profiles from Alg/Chit NPs were in accordance with targeted release doses that can vary from 20 to 200 ng/mL to control the GBM cell migration, depending on the initial loading concentration [23,41]. The release profiles were also in accordance with the release time frame anticipated for a future therapeutic approach (24 to 72 h) [46,47,48]. We found that the mechanism underlying this release was mainly diffusive, as determined by the Korsmeyer-Peppas model [39]. The calculated exponential coefficient value of ~0.41 is consistent with a predominant Fickian diffusion for a spherical geometry [39,40]. To the best of our knowledge, this is the first time that the release of CXCL12 from NPs is quantified using such a mechanistic approach. Mathematical modeling allowed us to estimate the effective diffusion coefficient (*D_eff_*) and the overall mass transfer coefficient (*k*). Interestingly, lower values (~2 × 10^−19^ m^2^/s) were obtained compared to what would be normally expected for a small protein such as CXCL12. Low diffusion coefficient values are consistent with our previous study involving a similar delivery system for the release of a small peptide derived from bone morphogenetic protein 9 for brain tissue applications [10]. As we have previously reported, low values of effective diffusion coefficient can be associated with the presence of electrostatic interactions between a positively charged protein/peptide, and the negatively charged Alg core composing the Alg/Chit NPs [10]. Hecq et al. have shown that for small proteins (insulin), Chit NPs leads to diffusion coefficients values that are several orders of magnitude lower than expected, as compared to macroscopic hydrogels filled with hydrophilic drugs [49]. In addition, the fact that not all of the CXCL12 molecules were released, combined with the lower than expected effective diffusion coefficient, strongly suggest the presence of electrostatic interactions between CXCL12 and Alg polymer chains. Indeed, weakly bound CXCL12 molecules diffused out of the NPs, from the core to the surface. Some of them, which have not already interacted with Alg chains, will see their release hindered as they make their way out of the NPs. As for the overall mass transfer coefficient “*k*”, similar values were obtained in our previous studies working with peptides released form Alg/Chit NPs [10] or collagen hydrogel [37]. Interestingly, no decrease in the parameter values was observed as the initial mass loading of CXCL12 increased, which indicates a decrease in the release rate. This may be caused by the presence of stronger interactions of CXCL12 with NPs and more CXCL12 aggregates formed while increasing the initial mass loading, as reported in other systems [38]. Therefore, when the proteins interact with the polymer network, interactions might hinder the diffusion of free proteins out of the Alg/Chit NPs, which is translated in our mathematical framework by a decrease in the “*k*” parameter value. The fact that the initial mass loading can affect the release rate offers a certain level of tunability. The delivery system can be designed in function of the CXCL12 dose required and GBM cell response to the chemoattractant gradient. As in vivo peri-tumoral environment should be harsher than controlled in vitro experiments, the presence of electrostatic interactions could provide a more prolonged release. For instance, following the completion of CXCL12 release by diffusion, other type of mass transport could occur due to NPs erosion or pH local changes as those NPs are pH sensitive [31]. In addition, since the release kinetic assays were conducted in static conditions, we also need to consider other phenomenon such as convective interstitial brain fluid flow, which could increase the release rate in vivo depending on the zone of the tumor. The fluidic phase of the brain parenchyma is considered to have both diffusive and convective contributions [50,51]. Slow release rate (low diffusion coefficient) and electrostatic interactions could be beneficial in this case and limit the release of the chemoattractant.

Furthermore, in the present study, the MTS assays did not reveal any significant effect of CXCL12 (free at 0.1, 0.4, or 0.8 µg/mL, or entrapped in the NPs) on the mitochondrial enzymatic activity in F98 cells in comparison to the untreated cells (CTL) after incubation for 24 h, suggesting that there was no cytotoxic effect of this chemokine or Alg/Chit NPs. Another experimental approach using live/dead cell staining assays confirmed these observations. Interestingly, the mitochondrial enzymatic activity in F98 cells incubated in the presence of Alg/Chit NPs without CXCL12 at 48 or 72 h was also similar to that observed in untreated cells, suggesting that the NPs alone did not promote the F98 cell proliferation. Indeed, we previously found that the Alg/Chit NPs alone can slightly enhance the proliferation of human neuroblastoma cells [10]. The proliferation of F98 cells at 48 or 72 h was also similar with or without CXCL12 (free or entrapped in NPs) treatment. However, several studies have shown that CXCL12 can increase the proliferation of GBM cells [41,52]. These apparent controversial results may be due not only to the different origins of the cells but also to the CXCL12 dose. For example, the proliferation of human U87-MG GBM cells was significantly increased in a dose dependent manner by human CXCL12 after incubation for 24 h, as shown by [^3^H] thymidine incorporation. Nevertheless, CXCL12 at a concentration of 12.5 nM, induced a higher DNA synthesis than at 50 nM [52]. In the same way, using [^3^H] thymidine incorporation, Bajetto et al. found that CXCL12 at 100 ng/mL promoted the proliferation of primary GBM cells obtained from a woman (36 years old), while it prevented it at 200 ng/mL [41]. In contrast, the proliferation of primary GBM cells obtained from a man (63 years old) was increased at 200 ng/mL [41]. These observations confirmed the importance to design a versatile CXCL12 delivery system.

Using Transwell^TM^ chemotaxis assays, the capacity of CXCL12 released from the Alg/Chit NPs to increase the invasion of F98 cells was assessed. Our results show that free CXCL12 significantly increased the invasion capacity of F98 cells compared to untreated cells. These results are in accordance with those of Rubin et al. [23]. Using 4-h Boyden chamber assays coated with laminin, they found that the maximal chemotaxis effect was reached at a CXCL12 concentration of 100 ng/mL [23]. Bajetto et al. also found using a Transwell^TM^ chemotaxis assay in a serum-free medium that the migration of human U87-MG GBM cells was significantly increased in a dose dependent manner by CXCL12 at a concentration varying from 1 to 50 ng/mL. There was a two-fold increase in cell migration at 50 ng/mL, compared to 1 ng/mL. However, even though the migration was still promoted with CXCL12 at 100 ng/mL compared to the control, it was lower than what had been observed at 50 ng/mL [41]. Such results highlight the importance to develop tunable CXCL12 delivery systems that can be modulated in the function of the GBM cell sensitivity. Our results also show that released CXCL12 from Alg/Chit NPs was able to significantly increase the migration of F98 cells through the layer of Matrigel. Wang et al. found an increase in the migration of mesenchymal stem cells in the presence of Chit/heparin NPs loaded with CXCL12 (5, 10 and 20 ng/mL) after incubation for 36 h. The migration was similar to that observed with free chemokine (5, 10, and 20 ng/mL). However, the authors did not characterize the CXCL12 release kinetics from NPs within 36 h to show the total delivery of the molecule. The comparison of released CXCL12 versus free chemokine is therefore difficult [25]. In the same way, Mansor et al. have found using an agarose drop migration assay that CXCL12 released from PLGA NPs (PLGA-COOH proportion (0%) allowing 75% CXCL12 encapsulation efficiency) can promote the migration of U87 MG GBM cells transfected to express CXCR4 receptors [26].

## 5. Conclusions

In this study, we have developed a tunable and efficient non-cytotoxic Alg/Chit NPs delivery system that can entrap various quantities of CXCL12 at high encapsulation efficiency within an applicable concentration range, while keeping its bioactivity. Alg/Chit NPs can also release CXCL12 at specific doses over time depending on the initial mass loading, to control GBM cell invasion without enhancing their proliferation. The release of CXCL12 has been shown to be primarily diffusive, but with strong interactions between the chemokine and the polymeric matrix. Analysis of the release kinetics and their mathematical modeling allow us to have certain control over the delivery process, which can be used to adapt the delivery to specific needs. We plan, in a future study, to deepen our knowledge on the tunability of this delivery system by evaluating its capacity to promote the attraction of GBM cells freshly isolated from human patients and which show different levels of aggressiveness. Indeed, as the possible clinical application of this delivery system may focus on personalized medicine, a pre-screening of GBM cell sensitivity prior to the use of the delivery system might be an interesting avenue. With this idea in mind, we will also further push our comprehension of the release mechanisms and adapt the mathematical framework consequently by more faithfully mimicking in vivo conditions using a 3D migration assay under perfusion flow. The relevant information thus obtained will help to optimize and adapt the therapeutic approach in vivo in regard to the location within the brain and the patient’s specific requirements. In this case, the injection of the NPs-CXCL12 may be performed in a second step, the first step being the tumor resection, allowing the access to GBM cells and evaluation of their sensitivity, followed by the implantation of the matrix/wafer adapted to the patient’s requirements to fill the surgical cavity.

## Figures and Tables

**Table 1:** Total mass encapsulated and encapsulation efficiency of the CXCL12 in Alg/Chit NPs (±STD).

Initial Mass Loading µg CXCL12/mg NPs	Total Mass Encapsulated µg CXCL12/mg NPs	Encapsulation Efficiency %
0.372	0.366 ± 0.003	98.5 ± 0.8
0.744	0.720 ± 0.005	96.6 ± 0.6
1.490	1.462 ± 0.016	98.1 ± 1.0

**Table 2:** Korsmeyer-Peppas model parameter estimation (±STD).

Initial Mass Loading µg CXCL12/mg NPs	Exponential Parameter
0.372	0.41 ± 0.01
0.744	0.37 ± 0.08
1.490	0.46 ± 0.15

**Table 3:** Mechanistic parameters estimation and statistics of the mathematical framework (±STD).

Initial Mass Loading µg CXCL12/mg NPs	*D_eff_* m^2^·s^−1^ × 10^19^	*k* m·s^−1^ × 10^9^	*R* ^2^	*p* Value	Lack of Fit
0.372	2.39 ± 1.13	269 ± 181	0.97 ± 0.02	2.63E-12	0.86
0.744	6.37 ± 0.61	20.5 ± 4.45	0.99 ± 0.01	1.48E-17	0.99
1.490	0.99 ± 0.23	4.72 ± 1.18	0.97 ± 0.02	1.59E-15	0.41

## References

[1] Mendes M., Sousa J.J., Pais A., Vitorino C. (2018). Targeted Theranostic Nanoparticles for Brain Tumor Treatment. Pharmaceutics.

[2] Nagpal K., Singh S.K., Mishra D.N. (2013). Optimization of brain targeted chitosan nanoparticles of Rivastigmine for improved efficacy and safety. Int. J. Biol. Macromol..

[3] Mohammed M.A., Syeda J.T.M., Wasan K.M., Wasan E.K. (2017). An Overview of Chitosan Nanoparticles and Its Application in Non-Parenteral Drug Delivery. Pharmaceutics.

[4] Elkadery A.A.S., Elsherif E.A., Ezz Eldin H.M., Fahmy I.A.F., Mohammad O.S. (2019). Efficient therapeutic effect of Nigella sativa aqueous extract and chitosan nanoparticles against experimentally induced Acanthamoeba keratitis. Parasitol. Res..

[5] Khor E., Lim L.Y. (2003). Implantable applications of chitin and chitosan. Biomaterials.

[6] Nicol S., Hosie G.W. (1993). Chitin production by krill. Biochem. Syst. Ecol..

[7] Jones M., Kujundzic M., John S., Bismarck A. (2020). Crab vs. Mushroom: A Review of Crustacean and Fungal Chitin in Wound Treatment. Mar. Drugs.

[8] Mohammed M.H., Williams P.A., Tverezovskaya O. (2013). Extraction of chitin from prawn shells and conversion to low molecular mass chitosan. Food Hydrocoll..

[9] Mi L., Liu H., Gao Y., Miao H., Ruan J. (2017). Injectable nanoparticles/hydrogels composite as sustained release system with stromal cell-derived factor-1alpha for calvarial bone regeneration. Int. J. Biol. Macromol..

[10] Lauzon M.-A., Marcos B., Faucheux N. (2018). Characterization of alginate/chitosan-based nanoparticles and mathematical modeling of their SpBMP-9 release inducing neuronal differentiation of human SH-SY5Y cells. Carbohydr. Polym..

[11] Kumar M.N.V.R., Muzzarelli R.A.A., Muzzarelli C., Sashiwa H., Domb A.J. (2004). Chitosan chemistry and pharmaceutical perspectives. Chem. Rev..

[12] Dalonneau F., Liu X.Q., Sadir R., Almodovar J., Mertani H.C., Bruckert F., Albiges-Rizo C., Weidenhaupt M., Lortat-Jacob H., Picart C. (2014). The effect of delivering the chemokine SDF-1alpha in a matrix-bound manner on myogenesis. Biomaterials.

[13] Richter R., Jochheim-Richter A., Ciuculescu F., Kollar K., Seifried E., Forssmann U., Verzijl D., Smit M.J., Blanchet X., von Hundelshausen P. (2014). Identification and characterization of circulating variants of CXCL12 from human plasma: Effects on chemotaxis and mobilization of hematopoietic stem and progenitor cells. Stem Cells Dev..

[14] Kondo K., Shintani S., Shibata R., Murakami H., Murakami R., Imaizumi M., Kitagawa Y., Murohara T. (2009). Implantation of adipose-derived regenerative cells enhances ischemia-induced angiogenesis. Arterioscler. Thromb. Vasc. Biol..

[15] Peled A., Petit I., Kollet O., Magid M., Ponomaryov T., Byk T., Nagler A., Ben-Hur H., Many A., Shultz L. (1999). Dependence of human stem cell engraftment and repopulation of NOD/SCID mice on CXCR4. Science.

[16] Kang H., Watkins G., Parr C., Douglas-Jones A., Mansel R.E., Jiang W.G. (2005). Stromal cell derived factor-1: Its influence on invasiveness and migration of breast cancer cells in vitro, and its association with prognosis and survival in human breast cancer. Breast Cancer Res..

[17] Mousavi A. (2020). CXCL12/CXCR4 signal transduction in diseases and its molecular approaches in targeted-therapy. Immunol. Lett..

[18] Guice E.D., Ford D.C. (1991). Developmental programs and remediation strategies in schools of nursing. NLN Publ..

[19] Stupp R., Hegi M.E., Mason W.P., van den Bent M.J., Taphoorn M.J.B., Janzer R.C., Ludwin S.K., Allgeier A., Fisher B., Belanger K. (2009). Effects of radiotherapy with concomitant and adjuvant temozolomide versus radiotherapy alone on survival in glioblastoma in a randomised phase III study: 5-year analysis of the EORTC-NCIC trial. Lancet. Oncol..

[20] McLendon R.E., Halperin E.C. (2003). Is the long-term survival of patients with intracranial glioblastoma multiforme overstated?. Cancer.

[21] Burger P.C., Dubois P.J., Schold S.C.J., Smith K.R.J., Odom G.L., Crafts D.C., Giangaspero F. (1983). Computerized tomographic and pathologic studies of the untreated, quiescent, and recurrent glioblastoma multiforme. J. Neurosurg..

[22] Goncalves R.M., Antunes J.C., Barbosa M.A. (2012). Mesenchymal stem cell recruitment by stromal derived factor-1-delivery systems based on chitosan/poly(gamma-glutamic acid) polyelectrolyte complexes. Eur. Cell. Mater..

[23] Rubin J.B., Kung A.L., Klein R.S., Chan J.A., Sun Y., Schmidt K., Kieran M.W., Luster A.D., Segal R.A. (2003). A small-molecule antagonist of CXCR4 inhibits intracranial growth of primary brain tumors. Proc. Natl. Acad. Sci. USA.

[24] Misra P., Lebeche D., Ly H., Schwarzkopf M., Diaz G., Hajjar R.J., Schecter A.D., Frangioni J.V. (2008). Quantitation of CXCR4 expression in myocardial infarction using 99mTc-labeled SDF-1alpha. J. Nucl. Med..

[25] Wang B., Tan L., Deng D., Lu T., Zhou C., Li Z., Tang Z., Wu Z., Tang H. (2015). Novel stable cytokine delivery system in physiological pH solution: Chitosan oligosaccharide/heparin nanoparticles. Int. J. Nanomedicine.

[26] Haji Mansor M., Najberg M., Contini A., Alvarez-Lorenzo C., Garcion E., Jerome C., Boury F. (2018). Development of a non-toxic and non-denaturing formulation process for encapsulation of SDF-1alpha into PLGA/PEG-PLGA nanoparticles to achieve sustained release. Eur. J. Pharm. Biopharm..

[27] Lauzon M.-A., Daviau A., Marcos B., Faucheux N. (2015). Nanoparticle-mediated growth factor delivery systems: A new way to treat Alzheimer’s disease. J. Control. Release.

[28] Haque S., Md S., Sahni J.K., Ali J., Baboota S. (2014). Development and evaluation of brain targeted intranasal alginate nanoparticles for treatment of depression. J. Psychiatr. Res..

[29] Drouin G., Couture V., Lauzon M.-A., Balg F., Faucheux N., Grenier G. (2019). Muscle injury-induced hypoxia alters the proliferation and differentiation potentials of muscle resident stromal cells. Skelet. Muscle.

[30] Ronneberger O., Fischer P., Brox T. U-Net: Convolutional Network for Biomedical Image Segmentation. Proceedings of the Medical Image Computing and Computer-Assisted Intervention—MICCAI 2015.

[31] Rahaiee S., Shojaosadati S.A., Hashemi M., Moini S., Razavi S.H. (2015). Improvement of crocin stability by biodegradeble nanoparticles of chitosan-alginate. Int. J. Biol. Macromol..

[32] Cardia M.C., Carta A.R., Caboni P., Maccioni A.M., Erbi S., Boi L., Meloni M.C., Lai F., Sinico C. (2019). Trimethyl Chitosan Hydrogel Nanoparticles for Progesterone Delivery in Neurodegenerative Disorders. Pharmaceutics.

[33] Bot N., Schweizer C., Ben Halima S., Fraering P.C. (2011). Processing of the synaptic cell adhesion molecule neurexin-3beta by Alzheimer disease alpha- and gamma-secretases. J. Biol. Chem..

[34] Emami J., Boushehri M.S.S., Varshosaz J. (2014). Preparation, characterization and optimization of glipizide controlled release nanoparticles. Res. Pharm. Sci..

[35] Goycoolea F.M., Lollo G., Remunan-Lopez C., Quaglia F., Alonso M.J. (2009). Chitosan-alginate blended nanoparticles as carriers for the transmucosal delivery of macromolecules. Biomacromolecules.

[36] Li P., Dai Y.-N., Zhang J.-P., Wang A.-Q., Wei Q. (2008). Chitosan-alginate nanoparticles as a novel drug delivery system for nifedipine. Int. J. Biomed. Sci..

[37] Lauzon M.-A., Marcos B., Faucheux N. (2014). Effect of initial pBMP-9 loading and collagen concentration on the kinetics of peptide release and a mathematical model of the delivery system. J. Control. Release.

[38] Wu J.-Y., Liu S.-Q., Heng P.W.-S., Yang Y.-Y. (2005). Evaluating proteins release from, and their interactions with, thermosensitive poly (N-isopropylacrylamide) hydrogels. J. Control. Release.

[39] Korsmeyer R.W., Gurny R., Doelker E. (1983). Mechanisms of solute release from porous hydrophilic polymers. Int. J. Pharm..

[40] Ritger P.L., Peppas N.A. (1987). A simple equation for description of solute release. J. Control. Release.

[41] Bajetto A., Barbieri F., Dorcaratto A., Barbero S., Daga A., Porcile C., Ravetti J.L., Zona G., Spaziante R., Corte G. (2006). Expression of CXC chemokine receptors 1–5 and their ligands in human glioma tissues: Role of CXCR4 and SDF1 in glioma cell proliferation and migration. Neurochem. Int..

[42] Mahmoud B.S., AlAmri A.H., McConville C. (2020). Polymeric Nanoparticles for the Treatment of Malignant Gliomas. Cancers.

[43] Jin G.-Z., Chakraborty A., Lee J.-H., Knowles J.C., Kim H.-W. (2020). Targeting with nanoparticles for the therapeutic treatment of brain diseases. J. Tissue Eng..

[44] Shin S.W., Song I.H., Um S.H. (2015). Role of Physicochemical Properties in Nanoparticle Toxicity. Nanomaterials.

[45] Zamproni L.N., Mundim M.V., Porcionatto M.A., des Rieux A. (2017). Injection of SDF-1 loaded nanoparticles following traumatic brain injury stimulates neural stem cell recruitment. Int. J. Pharm..

[46] Zhang J., Sarkar S., Yong V.W. (2005). The chemokine stromal cell derived factor-1 (CXCL12) promotes glioma invasiveness through MT2-matrix metalloproteinase. Carcinogenesis.

[47] Goffart N., Kroonen J., Di Valentin E., Dedobbeleer M., Denne A., Martinive P., Rogister B. (2015). Adult mouse subventricular zones stimulate glioblastoma stem cells specific invasion through CXCL12/CXCR4 signaling. Neuro Oncol..

[48] Ko C.-Y., Wu L., Nair A.M., Tsai Y.-T., Lin V.K., Tang L. (2012). The use of chemokine-releasing tissue engineering scaffolds in a model of inflammatory response-mediated melanoma cancer metastasis. Biomaterials.

[49] Hecq J., Siepmann F., Siepmann J., Amighi K., Goole J. (2015). Development and evaluation of chitosan and chitosan derivative nanoparticles containing insulin for oral administration. Drug Dev. Ind. Pharm..

[50] Han H., Li K., Yan J., Zhu K., Fu Y. (2012). An in vivo study with an MRI tracer method reveals the biophysical properties of interstitial fluid in the rat brain. Sci. China Life Sci..

[51] Nicholson C., Hrabetova S. (2017). Brain Extracellular Space: The Final Frontier of Neuroscience. Biophys. J..

[52] Barbero S., Bonavia R., Bajetto A., Porcile C., Pirani P., Ravetti J.L., Zona G.L., Spaziante R., Florio T., Schettini G. (2003). Stromal cell-derived factor 1alpha stimulates human glioblastoma cell growth through the activation of both extracellular signal-regulated kinases 1/2 and Akt. Cancer Res..

