# Erratum: Gascon S.; et al. Characterization and Mathematical Modeling of Alginate/Chitosan-Based Nanoparticles Releasing the Chemokine CXCL12 to Attract Glioblastoma Cells. *Pharmaceutics* 2020, *12*, 356

**DOI:** 10.3390/pharmaceutics12121153

**Published:** 2020-11-27

**Authors:** Suzanne Gascon, Angéla Giraldo Solano, Wiam El Kheir, Hélène Therriault, Pierre Berthelin, Bettina Cattier, Bernard Marcos, Nick Virgilio, Benoit Paquette, Nathalie Faucheux, Marc-Antoine Lauzon

**Affiliations:** 1 Laboratory of Cell-Biomaterial Biohybrid Systems, Department of Chemical and Biotechnological Engineering, Faculty of Engineering, Université de Sherbrooke, 2500 Boul. Université, Sherbrooke, QC J1K 2R1, Canada; Suzanne.Gascon@USherbrooke.ca (S.G.); Pierre.Berthelin@USherbrooke.ca (P.B.); Nathalie.Faucheux@USherbrooke.ca (N.F.); 2 Department of Nuclear Medicine and Radiobiology, Faculty of Medicine and Health Sciences, Université de Sherbrooke, 12e Avenue Nord, Sherbrooke, QC J1H 5N4, Canada; Angela.Giraldo.Solano@USherbrooke.ca (A.G.S.); Helene.Therriault@USherbrooke.ca (H.T.); Benoit.Paquette@USherbrooke.ca (B.P.); 3 Advanced Dynamic Cell Culture Systems Laboratory, Department of Chemical and Biotechnological Engineering, Faculty of Engineering, Université de Sherbrooke, 2500 Boul. Université, Sherbrooke, QC J1K 2R1, Canada; Wiam.El.Kheir@USherbrooke.ca (W.E.K.); b.cattier@hubebi.com (B.C.); 4 Department of Chemical and Biotechnological Engineering, Faculty of Engineering, Université de Sherbrooke, 2500 Boul. Université, Sherbrooke, QC J1K 2R1, Canada; Bernard.Marcos@USherbrooke.ca; 5 Department of Chemical Engineering, Polytechnique Montréal, Montréal, QC H3C 3A7, Canada; Nick.Virgilio@polymtl.ca; 6 Clinical Research Center of the Centre Hospitalier Universitaire de l’Université de Sherbrooke, 12e Avenue Nord, Sherbrooke, QC J1H 5N4, Canada; 7 Research Center on Aging, 1036, rue Belvédère Sud, Sherbrooke, QC J1H 4C4, Canada

**Keywords:** nanoparticles, chitosan, alginate, delivery system, mathematical modeling, chemokine, cell migration

The authors wish to make the following correction to this paper [1]: In the *Materials and Methods* Section, there is a missing term (*r²*) in Equation (3), which depicts Fick’s second law of diffusion for spherical coordinates in the radial dimension assuming a constant diffusion coefficient. The equation should have been written as follows:

(3) $$\frac{\partial C_{CXCL12}}{\partial t}=\frac{D_{eff}}{r^{2}}\left[\frac{\partial }{\partial r}\left(r^{2}\frac{\partial C_{CXCL12}}{\partial r}\right)\right]$$

Note that this typographical error does not affect in any way the results or conclusions of the article.

The authors would like to apologize for any inconvenience caused to the readers by these changes.
